# Assessment of the cardiovascular adverse effects of drug-drug interactions through a combined analysis of spontaneous reports and predicted drug-target interactions

**DOI:** 10.1371/journal.pcbi.1006851

**Published:** 2019-07-19

**Authors:** Sergey Ivanov, Alexey Lagunin, Dmitry Filimonov, Vladimir Poroikov

**Affiliations:** 1 Department of Bioinformatics, Institute of Biomedical Chemistry, Moscow, Russia; 2 Medico-biological Faculty, Pirogov Russian National Research Medical University, Moscow, Russia; University of Chicago, UNITED STATES

## Abstract

Adverse drug effects (ADEs) are one of the leading causes of death in developed countries and are the main reason for drug recalls from the market, whereas the ADEs that are associated with action on the cardiovascular system are the most dangerous and widespread. The treatment of human diseases often requires the intake of several drugs, which can lead to undesirable drug-drug interactions (DDIs), thus causing an increase in the frequency and severity of ADEs. An evaluation of DDI-induced ADEs is a nontrivial task and requires numerous experimental and clinical studies. Therefore, we developed a computational approach to assess the cardiovascular ADEs of DDIs. This approach is based on the combined analysis of spontaneous reports (SRs) and predicted drug-target interactions to estimate the five cardiovascular ADEs that are induced by DDIs, namely, myocardial infarction, ischemic stroke, ventricular tachycardia, cardiac failure, and arterial hypertension. We applied a method based on least absolute shrinkage and selection operator (LASSO) logistic regression to SRs for the identification of interacting pairs of drugs causing corresponding ADEs, as well as noninteracting pairs of drugs. As a result, five datasets containing, on average, 3100 potentially ADE-causing and non-ADE-causing drug pairs were created. The obtained data, along with information on the interaction of drugs with 1553 human targets predicted by PASS Targets software, were used to create five classification models using the Random Forest method. The average area under the ROC curve of the obtained models, sensitivity, specificity and balanced accuracy were 0.837, 0.764, 0.754 and 0.759, respectively. The predicted drug targets were also used to hypothesize the potential mechanisms of DDI-induced ventricular tachycardia for the top-scoring drug pairs. The created five classification models can be used for the identification of drug combinations that are potentially the most or least dangerous for the cardiovascular system.

## Author summary

Assessment of adverse drug effects as well as the influence of drug-drug interactions on their manifestation is a nontrivial task that requires numerous experimental and clinical studies. We developed a computational approach for the prediction of adverse effects that are induced by drug-drug interactions, which are based on a combined analysis of spontaneous reports and predicted drug-target interactions. Importantly, the approach requires only structural formulas to predict adverse effects, and, therefore, may be applied for new, insufficiently studied drugs. We applied the approach to predict five of the most important cardiovascular adverse effects, because they are the most dangerous and widespread. These effects are myocardial infarction, ischemic stroke, ventricular tachycardia, arterial hypertension and cardiac failure. The accuracies of predictive models were relatively high, in the range of 73–81%; therefore, as example, we performed a prediction of the five cardiovascular adverse effects for the large number of drug pairs and revealed the combinations that may potentially cause ventricular tachycardia along with potential molecular mechanisms. We consider that the developed approach can be used for the identification of pairwise drug combinations that are potentially the most or least dangerous for the cardiovascular system.

## Introduction

Adverse drug effects (ADEs) are one of the top 10 causes of death in developed countries, are one of the main reasons for stopping the development of new drug-candidates and are the main reason for drug recalls from the market [1, 2]. Cardiovascular effects are some of the most serious ADEs that may lead to hospitalization or death, and, at the same time, are widespread [1]. The ADE profile of a particular drug-candidate is usually investigated during standard preclinical animal tests and clinical trials according to the GLP and GCP requirements. However, many rare, but serious, ADEs cannot be revealed by these studies, because of interspecies differences, the limited number of patients or animals and the duration of studies; thus, additional *in vitro* and *in silico* methods for the detection of serious ADEs are currently being developed [3–8]. These methods are based on the determination of the relationships between several chemical and biological features of drugs and their ADEs. Among these features are molecular descriptors, known and predicted drug targets, gene expression changes induced by drugs, phenotypic features such as perturbed pathways, or known ADEs. The relationships between these features and ADEs are usually established using various machine learning methods and network-based approaches. It is accepted that the interaction with human proteins is the most common cause of ADEs; therefore, known and predicted human targets are the most common type of drug features that are used in corresponding studies. Many of the developed methods require knowledge of only the structural formula of a drug-candidate to predict its potential ADEs; therefore, they can be used at the earliest stages of drug development, which may sufficiently increase their effectiveness [3, 4, 8].

In real clinical practice, the treatment of human diseases often requires the administration of several drugs, which can lead to drug-drug interactions (DDIs), thus causing an increase in the frequency and severity of ADEs [9]. An evaluation of the effect of DDIs on the manifestation of ADEs is a nontrivial task and requires numerous preclinical and clinical studies. To solve this problem various computational approaches for the prediction of DDIs were developed [10–22]. Most of these approaches are based on the calculation of similarities between the profiles of various chemical and biological features of two drugs. These similarities can be calculated based on molecular fingerprints, drug targets, their amino acid sequences, pathways and Gene Ontology (http://www.geneontology.org/) annotations, the Anatomical Therapeutic Chemical (ATC) Classification terms (https://www.whocc.no/atc_ddd_index/), as well as known ADEs of individual drugs [10, 12, 13, 15–17, 18, 20, 22]. The Tanimoto coefficient is the most common similarity that is measure in these studies; however, more complicated measures can be used, e.g., several approaches were developed to calculate the proximity of the protein targets of two drugs in a protein-protein interaction network [12, 17]. Similarity measures based on the profiles of different features can be integrated into single interaction scores that allow drug pairs to be ranked according to their potential ability to interact with each other. To estimate the parameters of such integration and validation of obtained results, information about known DDIs was used. Such data can be obtained from various public databases, including DrugBank (https://www.drugbank.ca/). For example, Cheng F. with colleagues [13] used several machine learning methods with drug phenotypic, therapeutic, chemical and genomic similarities used as features to predict DDIs. The classifiers were trained on the set of known DDIs from the DrugBank database and the same number of randomly chosen drug pairs as the negative examples. The best result with the area under the ROC-curve (AUC) 0.67 was achieved using a support vector machine with a Gaussian radial basis function kernel. In addition to approaches that are based on similarities, some other methods were developed [14, 19]. Zakharov A.V. with colleagues [19] used separate training sets of pairwise drug combinations for each of four isoforms of cytochromes P450, which are examples of known DDIs. The corresponding information was obtained from the literature. Drug pairs were represented as mixtures of compounds in ratio 1:1, and several types of molecular descriptors were generated for them. The prediction models were generated by using the radial basis function self-consistent regression and a Random Forest. The balanced accuracies that were obtained from the cross-validation procedure varied from 0.72 to 0.79, depending on the dataset [19]. Luo H., with colleagues, used the sums and differences of the docking scores for 611 human proteins to describe 6328 drug pairs, which represented known DDIs from the DrugBank database, and the same number of drug pairs was randomly chosen as a negative example. A predictive model was created based on l2-regularized logistic regressions to obtain their values. The obtained accuracy, sensitivity and specificity that were calculated based on the 10-fold cross-validation procedure were 0.804, 0.847 and 0.772, respectively [14].

Despite the significant progress in predicting DDIs, all of these methods allow for estimating only the fact of interaction, but not the resulting ADEs, whereas such information is important to assess the clinical significance of DDIs. The main problem is the absence of known data for most of the DDI-induced ADEs. The major source of data on ADEs of individual drugs is drug labels [23]; however, they usually contain very few data on ADEs that are induced by DDIs. Nevertheless, the corresponding information can be obtained through the analysis of spontaneous reports (SRs) which are received by regulatory agencies from healthcare professionals and patients. Each SR contains information about all drugs that are prescribed to a patient, as well as information about developed ADEs. An analysis of large sets of SRs allows for relationships between certain ADEs and individual drugs [24–29], or drug combinations [30–35], to be revealed. The datasets of individual drugs with information about ADEs obtained by an analysis of SRs were earlier successfully used for the creation of predictive models that are based on structure-activity relationships [27, 29]. The corresponding information on ADEs that is induced by pairwise drug combinations may also potentially be used for this purpose.

We developed a computational approach for the assessment of cardiovascular ADEs of DDIs. The approach is based on a combined analysis of SRs and predicted drug-target interactions (DTIs) and allows for the prediction of five cardiovascular ADEs of DDIs: myocardial infarction, ischemic stroke, ventricular tachycardia, arterial hypertension and cardiac failure, with balanced accuracies from 0.73 to 0.81. Unlike most of the other methods, our approach requires only structural formulas to predict cardiovascular adverse effects for any pair of drugs, and, therefore, may be applied for new, drug-like compounds that have not yet been studied. The developed approach can be used for the identification of pairwise drug combinations that are potentially the most or least dangerous for the cardiovascular system.

## Results and discussion

### General description of the approach

**Fig 1:**
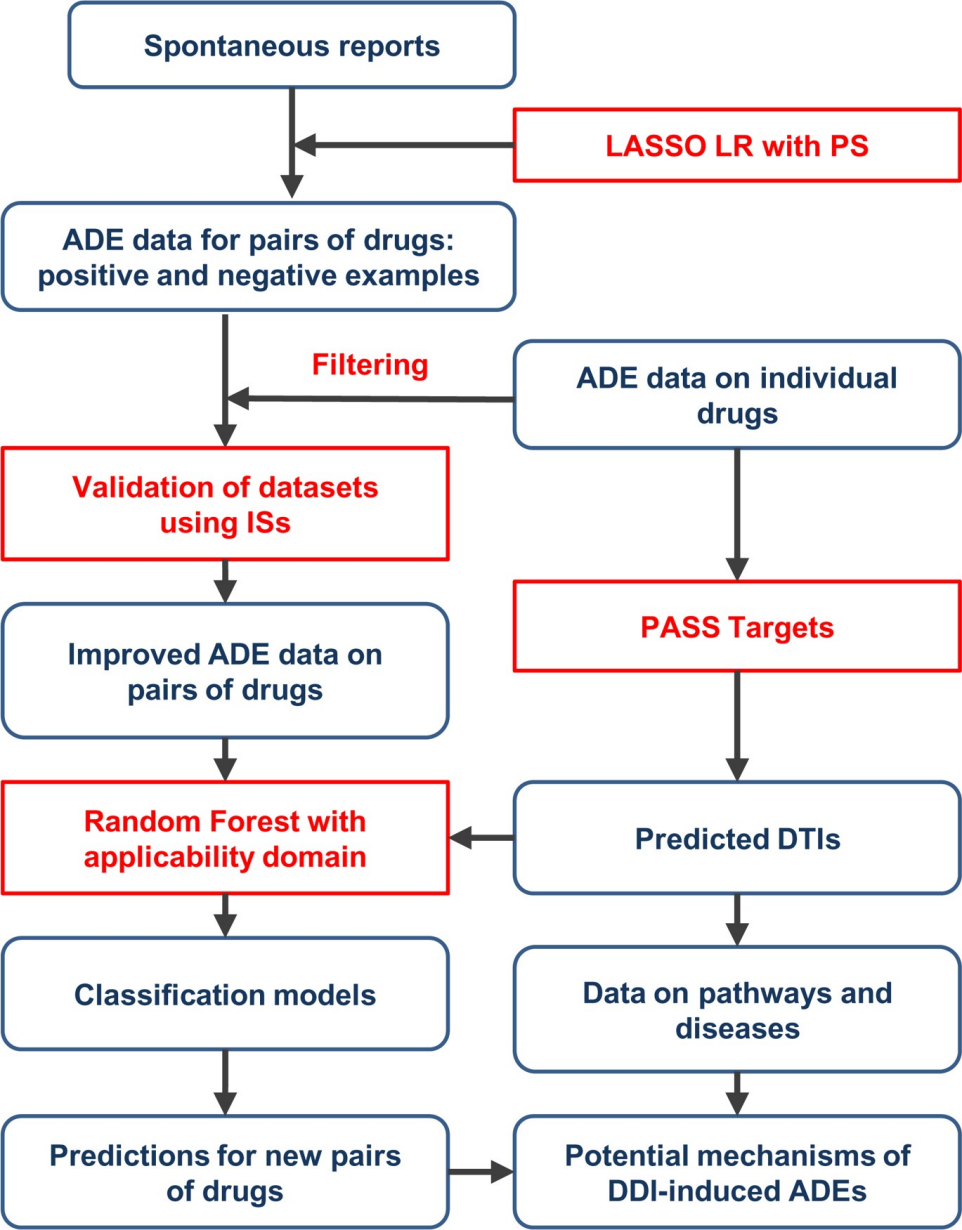
The scheme of a developed computational approach for the assessment of cardiovascular ADEs of DDIs. ISs–inference scores from Comparative Toxicogenomics Database, LASSO LR–least absolute shrinkage and selection operator (LASSO) logistic regression, PS–propensity scores (see Material and Methods).

We developed a new computational approach for the assessment of cardiovascular ADEs of DDIs through a combined analysis of SRs and predicted DTIs (Fig 1).

The approach is based on two main steps: creation of datasets on cardiovascular DDI-induced ADEs containing drug pairs that potentially cause or do not cause ADEs, and the creation of classification models for each dataset based on predicted drug targets as descriptors. The creation of datasets is based on the analysis of SRs from the standardized version of publicly available parts of the FDA database [36]. The analysis was performed using least absolute shrinkage and selection operator (LASSO) logistic regression with the addition of propensity scores as independent variables [35] (see Materials and Methods for details), which allows for the identification of drug pairs that potentially cause or do not cause cardiovascular ADEs–positive and negative examples. Each “positive” drug pair represents a potential synergistic or additive effect of DDI on the development of ADEs. This method takes into account the confounding effects of other drugs and risk factors on the manifestation of ADEs and, thus, allows for datasets with lower numbers of false positives to be obtained. To further improve the quality of datasets, information about the ADEs of individual drugs [37] was used to filter out potentially false positive and false negative examples (see Materials and Methods). Since the created datasets may still contain non-causal drug pair-ADE associations, we used an approach based on inference scores (ISs) [38] derived from Comparative Toxicogenomics Database (http://ctdbase.org/) to validate them and estimate their quality (see Materials and Methods).

At the second step of the approach, a PASS Targets software [39] was used to predict interactions of individual drugs that were from obtained datasets with 1553 human protein targets. The sums and absolute values of the differences in the probability estimates of interaction with targets were used as descriptors for drug pairs. The classification models were built using Random Forest along with a method that allows for the applicability domain to be determined. The accuracy of prediction is estimated using a 5-fold cross-validation procedure (see Materials and Methods). To demonstrate the practical benefit of the obtained models, predictions of ADEs for a large amount of drug pairs were performed. The analysis of the biological role of predicted protein targets for the top predicted drug pairs that potentially cause ADEs allows for proposing the potential mechanisms of corresponding DDIs.

### Creation of datasets and their validation

At the first step of the proposed approach, we created five datasets of drug pairs that potentially cause and do not cause five cardiovascular ADEs through the analysis of SRs (see Materials and Methods), namely, ventricular tachycardia, myocardial infarction, ischemic stroke, arterial hypertension and cardiac failure (see S1 Table). Each positive drug pair represents an example of a potential synergistic or additive DDI that causes a corresponding ADE. The datasets contain, on average, more than 3100 drug pairs belonging to 335 individual drugs and 166 ATC terms (https://www.whocc.no/atc_ddd_index/) of the fourth level (Table 1), reflecting the chemical/therapeutic/pharmacological subgroup of drugs, which indicates that the created datasets are representative.

**Table 1:** Characteristics of created datasets on potential DDI-induced ADEs.

	Positive pairs	Negative pairs	Number of drugs	Number of ATC classes
Ventricular tachycardia	933	2912	376	181
Myocardial infarction	2479	1279	352	168
Ischemic stroke	838	2101	331	169
Arterial hypertension	549	1029	273	146
Cardiac failure	1350	2108	343	166

Since the datasets were created by analysis of SRs and were not confirmed experimentally, they may still contain non-causal associations between drug pairs and ADEs. To validate them, we used a method based on inference scores (ISs) [38] from Comparative Toxicogenomics Database (http://ctdbase.org/). ISs are calculated from known drug-gene-disease relationships and reflect the influence of drugs on disease manifestation (therapeutic or adverse effect) (see Materials and Methods). We compared ISs for corresponding diseases between drug pairs from created datasets, which potentially cause and do not cause cardiovascular ADEs. We calculated AUC values for each dataset and p-values based on the Wilcoxon test to estimate their statistical significance (Table 2).

**Table 2:** The area under the ROC curve values and their statistical significance calculated for the created datasets based on inference scores.

	AUC	p-value
Arterial hypertension	0.901	2.20E-16
Ventricular tachycardia	0.760	2.20E-16
Cardiac failure	0.758	2.20E-16
Myocardial infarction	0.715	2.20E-16
Ischemic stroke	0.615	2.20E-16

The corresponding values range from 0.901 to 0.615 and reflect the quality of the datasets. According to AUC values, the dataset for arterial hypertension has the best quality, whereas the dataset for ischemic stroke has the worst quality. It is important to note that AUC values reflect both errors in datasets, caused by disadvantages of the analysis of SRs, and errors of approach, which was used for the calculation of corresponding ISs. Thus, the AUC values reflecting the quality of datasets must really be higher.

According to the obtained results, we can conclude that the created datasets have from good to moderate quality and can be used for further analysis.

### Prediction of DDI-induced cardiovascular ADEs based on drug-target interactions

We used Random Forest to create classification models based on five datasets and the local (Tree) approach to determine their applicability domain [40]. The models were created based on sums and absolute values of differences of probability estimates of interaction with 1553 human protein targets that had been calculated for individual drugs by PASS Targets software [39]. The accuracy estimates were obtained by a 5-fold cross-validation procedure with use of the “compound out” approach [41] (see Materials and Methods for details). The obtained average values of AUC, sensitivity, specificity and balanced accuracy were 0.837, 0.764, 0.754 and 0.759, respectively, whereas 95.7% of the drug pairs were in the applicability domain of the models (Table 3). The accuracy values generally correlate with the AUC values obtained using ISs (Table 2).

**Table 3:** Prediction accuracy for five cardiovascular DDI-induced ADEs based on 5-fold cross-validation procedure.

	AUC	Sensitivity	Specificity	Balanced accuracy	In applicability domain
Ventricular tachycardia	0.807	0.743	0.718	0.731	96.1%
Myocardial infarction	0.856	0.794	0.763	0.778	95.3%
Ischemic stroke	0.808	0.734	0.724	0.729	95.6%
Arterial hypertension	0.892	0.789	0.832	0.810	95.5%
Cardiac failure	0.824	0.761	0.734	0.747	96.1%
**Average**	**0.837**	**0.764**	**0.754**	**0.759**	**95.7%**

We also estimated the prediction accuracy of ventricular tachycardia and arterial hypertension on two external test sets, which are based on the data from the DrugBank database (see Materials and Methods) (Table 4).

**Table 4:** Prediction accuracy for ventricular tachycardia and arterial hypertension on external test sets.

	AUC	Sensitivity	Specificity	Balanced accuracy	In applicability domain
Ventricular tachycardia	0.779	0.865	0.519	0.692	80.3%
Arterial hypertension	0.779	0.741	0.748	0.744	73.8%

The obtained relatively high accuracies (Tables 3 and 4) allow for the application of the created models to solve practical tasks, e.g., to perform a search of new pairwise combinations of drugs that potentially interact and cause cardiovascular ADEs.

### Prediction of DDI-induced ADEs for the new drug pairs

The created datasets contain from hundreds to thousands of drug pairs that potentially cause cardiovascular ADEs depending on the effect; however, the number of possible pairwise drug combinations is much higher. To investigate the practical benefit of the created classification models, we performed a prediction of the DDIs-induced ADEs for all of the possible drug pairs that were generated from individual drugs with known data on five cardiovascular ADEs (see Materials and Methods) [37]. Five large datasets were generated with more than 230000 drug pairs on average, and 190000 pairs (84%) of them were in the applicability domain of the models (see Table 5).

**Table 5:** Numbers of drug pairs with predicted ADEs.

	Number of pairs	Number of pairs in AD	Pairs with ADE (P > 0.5)	Pairs with ADE (P > 0.8)
Ventricular tachycardia	279283	231438 (82.9%)	121486 (52.5%)	2885 (1.2%)
Myocardial infarction	235328	195688 (83.2%)	115399 (58.9%)	12397 (6.3%)
Ischemic stroke	223862	189334 (84.6%)	79031 (41.7%)	2176 (1.1%)
Arterial hypertension	187842	162784 (86.7%)	54744 (33.6%)	2994 (1.8%)
Cardiac failure	232183	193083 (83.1%)	94075 (48.7%)	4707 (2.4%)
**Average**	**231700**	**194465 (83.9%)**	**92947 (47.8%)**	**5032 (2.6%)**

**Fig 2:**
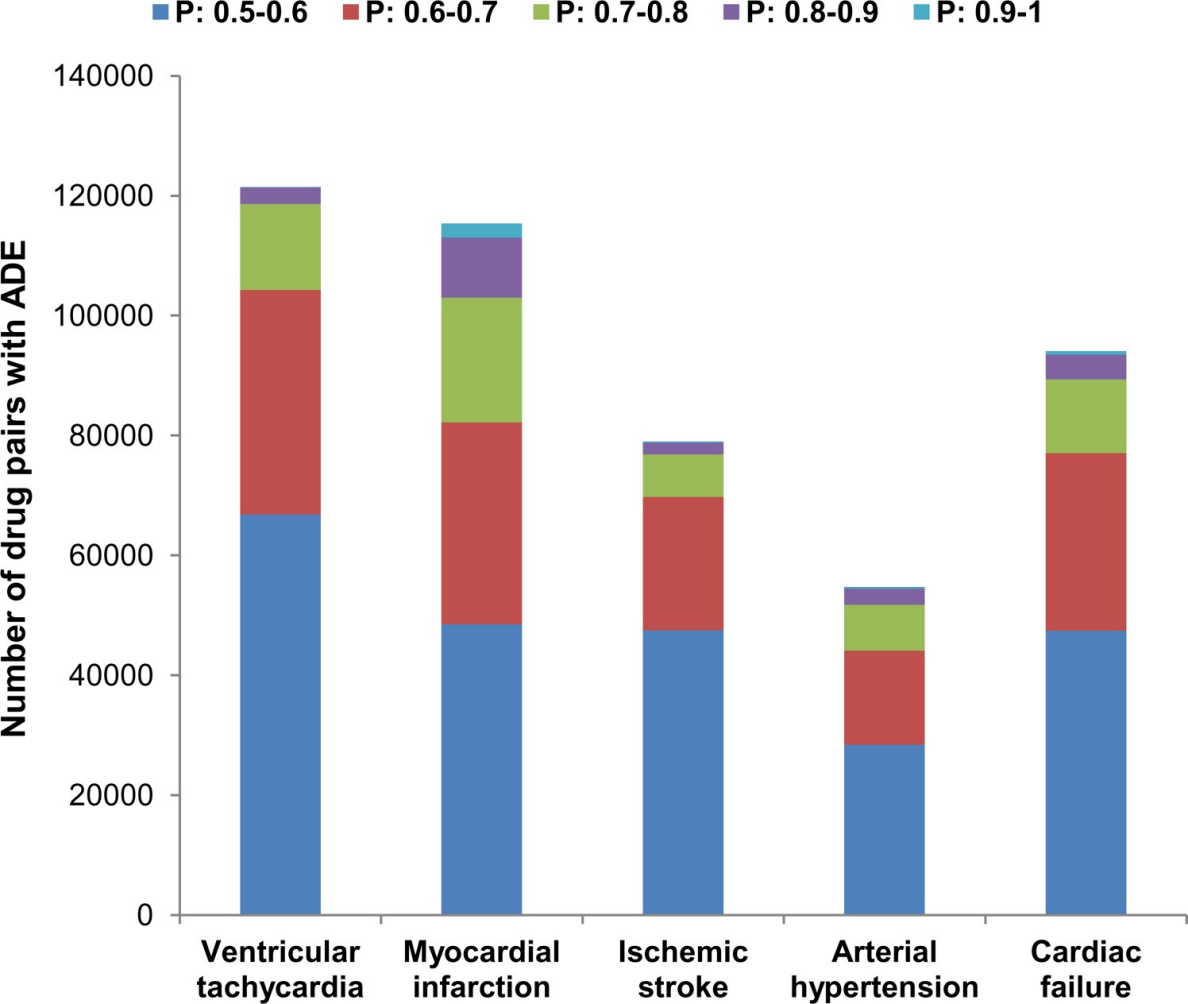
Distribution of predicted probabilities for five cardiovascular ADEs on large datasets of drug pairs.

Surprisingly, nearly half of the drug pairs in the datasets were predicted to cause corresponding DDI-induced ADEs. A large number of predicted drug pairs can be explained by a prediction probability distribution (Fig 2). Most of the predicted drug pairs have probability estimates are near the threshold P > 0.5, and they are unlikely to cause ADEs, whereas there are near 2.6% of drug pairs potentially cause ADEs at probability threshold P > 0.8 (Table 5).

**Fig 3:**
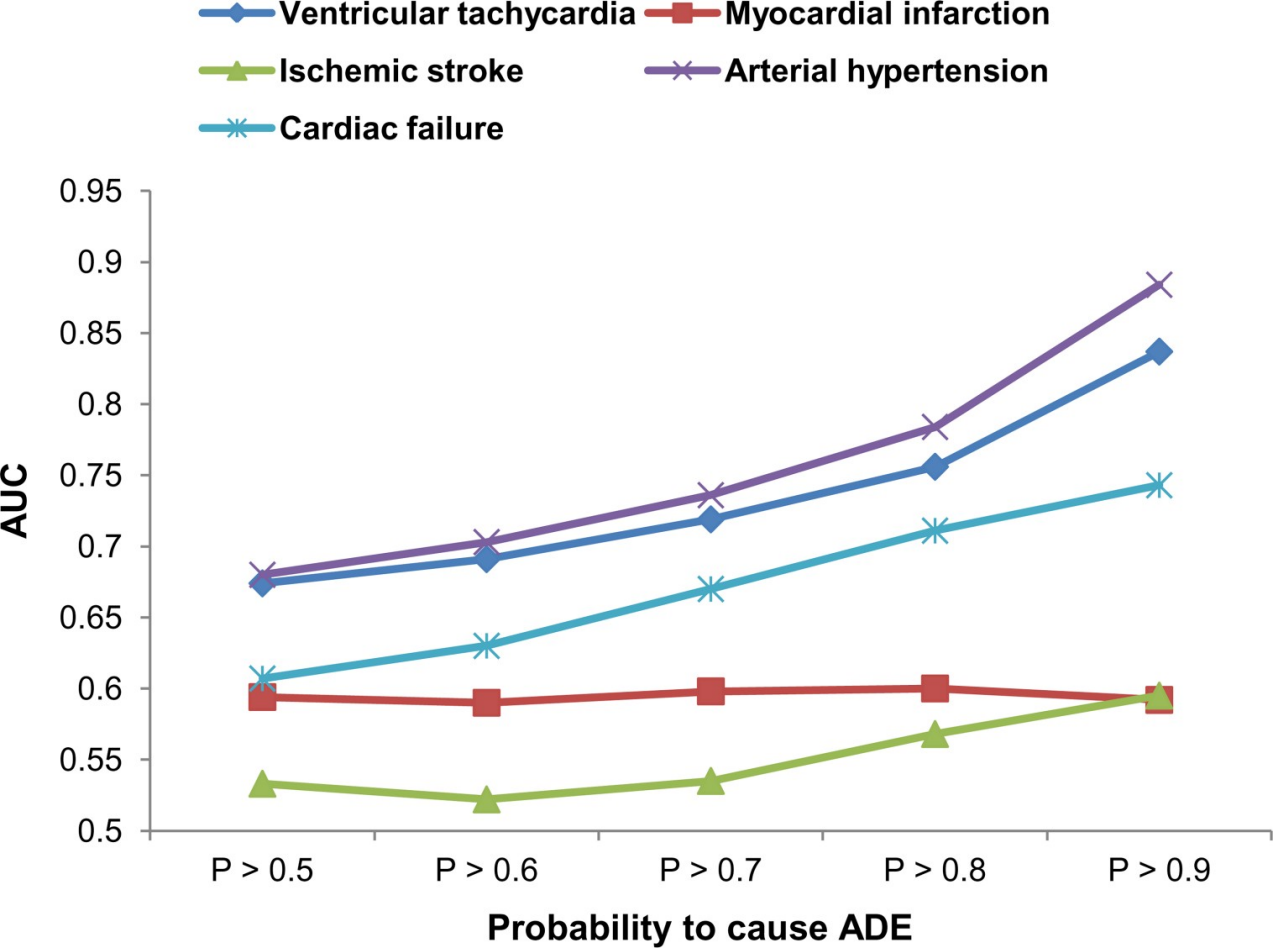
The area under the ROC curve values calculated based on inference scores at different thresholds of probabilities for large datasets.

To roughly estimate the accuracy of predictions for large datasets, we calculated AUC values based on ISs from Comparative Toxicogenomics Database at different thresholds of probabilities (Fig 3).

Fig 3 demonstrates that the AUC values for most of ADEs increase with increasing the probability threshold. The obtained AUC values at high probability thresholds are near the corresponding values obtained on training sets (see Table 2). Thus, high probability thresholds should be chosen for the selection of drug pairs potentially causing ADEs.

The results of these analyses and the results of 5-fold cross-validation (the average area under the ROC curve, sensitivity, specificity and balanced accuracy were 0.837, 0.764, 0.754 and 0.759, respectively; see Table 3) indicate that the accuracy of the prediction of the most of DDI-induced cardiovascular ADEs is relatively high and that the created models can be applied in the search for new pairwise combinations of drugs that are the most or the least dangerous for the cardiovascular system. Because DTIs are needed for the creation of models that were predicted by PASS Targets software based on structures of drugs, the developed models can be used for any drug-like compounds, including those for which only structural formulas are known. For example, they can be used to predict DDI-induced ADEs for drug candidates on the stage of clinical trials.

### Assessment of the potential mechanisms of DDI-induced ADEs

Since DDI-induced ADEs are effectively estimated by using data on predicted DTIs, the corresponding information on drug targets may also be used to reveal the potential mechanisms of cardiovascular ADEs and influence of DDIs on their manifestation.

We performed a corresponding analysis for the top 10 drug pairs from the large dataset with the highest probability scores for ventricular tachycardia (Table 6). We selected only those pairs where corresponding drugs do not cause ventricular tachycardia when administrated separately. According to prediction results, the drugs possibly cause ventricular tachycardia when they are administered together.

**Table 6:** Potential mechanisms of DDI-induced ventricular tachycardia for the top 10 scored drug pairs. The bold and underlined gene names mean known, experimentally confirmed drug targets from DrugBank and DrugCentral (http://drugcentral.org/) databases. Symbols ↑ and ↓ mean up- and down-regulation of the protein function by the drug.

Drug pairs	Common cytochromes P450	Known and predicted drug targets associated with ventricular tachycardia
Eszopiclone-Chlorphenamine	**CYP3A4**	Eszopiclone: **TSPO****↑**, CAMKK1, ULK1. Chlorphenamine: **HRH1****↓**, **SLC6A2****↓**, **HTR2B**, HRH2, KCNH2, CALM
Oxytetracycline-Temazepam	-	Temazepam: CAMKK1, CAMKK2, CAMK2A, **TSPO**
Nisoldipine-Chlorphenamine	**CYP3A4**, **CYP3A5**, **CYP3A7**	Nisoldipine: **CACNA1C****↓**, NR4A1. Chlorphenamine: **HRH1****↓**, **SLC6A2****↓**, **HTR2B**, HRH2, KCNH2, CALM
Tobramycin-Temazepam	-	Temazepam: CAMKK1, CAMKK2, CAMK2A, **TSPO**
Amikacin-Temazepam	-	Temazepam: CAMKK1, CAMKK2, CAMK2A, **TSPO**
Tetracycline-Temazepam	**CYP3A4**	Temazepam: CAMKK1, CAMKK2, CAMK2A, **TSPO**
Reboxetine-Chlorphenamine	**CYP2D6**, **CYP3A4**	Reboxetine: **SLC6A2****↓**. Chlorphenamine: **HRH1****↓**, **SLC6A2****↓**, **HTR2B**, HRH2, KCNH2, CALM
Alfentanil-Temazepam	**CYP3A4**	Alfentanil: KCNH2. Temazepam: CAMKK1, CAMKK2, CAMK2A, **TSPO**
Lovastatin-Guaifenesin	-	Lovastatin: **SLC6A2**. Guaifenesin: CALM, CAMKK2, SGK3
Celiprolol-Chlorphenamine	-	Celiprolol: **ADRA2A****↓**, **ADRA2B****↓**, **ADRA2C****↓**, **ADRB2****↑**, ULK1. Chlorphenamine: **HRH1****↓**, **SLC6A2****↓**, **HTR2B**, HRH2, KCNH2, CALM

We found that the DDIs for these drug pairs may occur at both levels of pharmacokinetics and pharmacodynamics. First, the drugs from five of ten pairs are metabolized by the same cytochromes P450. Second, corresponding drugs potentially interact with protein targets to influence the action potential of cardiac cells. These targets, either known or predicted, are shown in Table 6.

**Fig 4:**
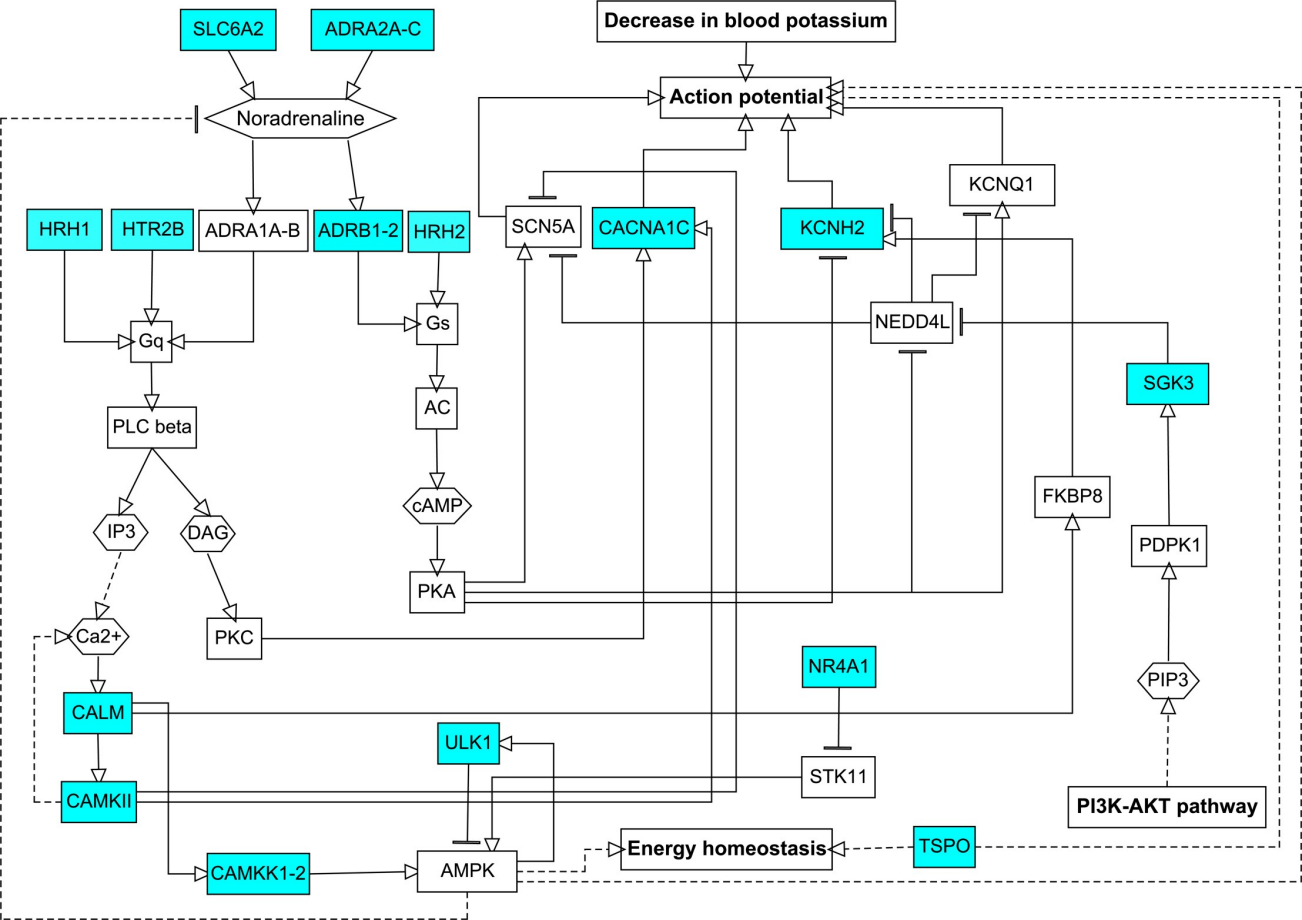
Influence of known and predicted protein targets of the top 10 scored drug pairs on the action potential in the heart. VT—ventricular tachycardia. Cyan nodes represent known and predicted protein targets of drugs from selected pairs, and white nodes represent intermediate proteins in the regulatory network. Solid edges represent direct interactions, and dashed edges represent indirect interactions. The figure was created based on data from KEGG pathways (https://www.genome.jp/kegg/pathway.html) and from corresponding information in the literature.

It is important that only chlorphenamine and alfentanil were predicted to interact with the HERG (KCNH2) potassium channel, which is a well-known protein that is associated with ventricular tachycardia [5]. However, this and other drugs from selected pairs that are known to or are predicted to interact with human proteins form compact fragments of the regulatory network (Fig 4) and indirectly change the action potential. Such changes may form a basis for the induction of ventricular tachycardia in predisposed patients.

## Materials and methods

### Data on cardiovascular ADEs of individual drugs

The data on cardiovascular ADEs of individual drugs were obtained from our previous study [37]. Briefly, we created five datasets of individual drugs which cause and do not cause the following cardiovascular ADEs: ventricular tachycardia, myocardial infarction, ischemic stroke, arterial hypertension, and cardiac failure. The primary source of information for the creation of datasets was SIDER 4.1 (http://sideeffects.embl.de/), which contains data on ADEs of drugs obtained from drug labels [23]. For each drug-ADE pair, we manually checked the section of the drug label where the ADE was described. If it was described in “Boxed Warning” or “Warnings and Precautions” sections, we considered that drug causes ADE. If ADE was described in section “Adverse reactions,” which may contain effects unrelated to drug intake, it had to be verified. To do this, additional information on ADEs was obtained using the following sources and approaches:

- spontaneous reports (SRs) and electronic medical records. To identify potential relationships between drugs and ADEs, disproportionality analysis was performed (see the publication [37] for details);
- Comparative Toxicogenomics Database (http://ctdbase.org/) which contains information about ADEs obtained from the literature.

We considered drug-ADE association from “Adverse reactions” section to be verified if it was confirmed from at least one additional source. If ADE was not indicated in the drug labels and publications although the compound had been used clinically for > 5 years and had > 50 SRs about other effects, then it was considered not to cause the corresponding effect. We proposed that integration of information from various sources allow filtering out most of false positive and false negative drug-ADE associations from created datasets.

### Assessment of DDI-induced ADEs through the analysis of SRs

In our current study, we used the AEOLUS database [36] as a source of SRs. AEOLUS is a curated version of publicly available parts of the FDA database of SRs (https://www.fda.gov/Drugs/GuidanceComplianceRegulatoryInformation/Surveillance/AdverseDrugEffects/default.htm), where the names of ADEs, drugs and indications are standardized. We selected only those SRs that contain description of drugs, ADEs and drug indications, because all of these types of data are required for further analysis. A total of 4028051 SRs were selected. The ADEs and indications in the database were described by the preferred terms (PTs) of the MedDRA dictionary (https://www.meddra.org/). Since some PTs may describe pathologies that are related to the same or similar ADEs, we selected the main PTs, which exactly match the investigated ADEs and supporting PTs, which are conditions that are similar to or are indirectly related to ADEs. The main and supporting PTs for five investigated cardiovascular ADEs are presented in Table 7.

**Table 7:** Main and supporting PTs for five investigated cardiovascular ADEs.

Main PTs	Supporting PTs
Torsade de Pointes Ventricular Tachycardia	Electrocardiogram QT Prolonged Electrocardiogram QT Corrected Interval Prolonged Ventricular Arrhythmia
Acute Myocardial Infarction Acute Coronary Syndrome Myocardial Infarction	Angina Pectoris Angina Unstable Arteriosclerosis Coronary Artery Arteriospasm Coronary Coronary Artery Disease Coronary Artery Occlusion Coronary Artery Stenosis Coronary Artery Thrombosis Myocardial Ischemia
Hypertension Hypertensive Crisis	Blood Pressure Increased Blood Pressure Systolic Increased Blood Pressure Diastolic Increased
Cerebrovascular Accident Cerebral Infarction Ischemic Stroke	Cerebral Ischemia Transient Ischemic Attack
Cardiac Failure Acute Cardiac Failure Congestive Cardiac Failure Cardiogenic Shock Cardiopulmonary Failure Left Ventricular Failure Right Ventricular Failure	–

At the next step, we selected those drugs in the AEOLUS database that have annotations on five investigated cardiovascular ADEs: ventricular tachycardia, myocardial infarction, ischemic stroke, arterial hypertension and cardiac failure. The data on drugs that caused and did not cause five ADEs was obtained from our previous study [37] (see above). The following numbers of drugs were selected: 496 drugs for ventricular tachycardia, 460 drugs for myocardial infarction, 447 drugs for ischemic stroke, 398 drugs for arterial hypertension, and 467 drugs for cardiac failure. The data on the five ADEs of these individual drugs are represented in S2 Table.

We selected drug pairs that were formed by these drugs with at least 100 SRs wherein both drugs are mentioned. For each pair of drugs and each PT from Table 7, we performed an analysis which is based on three steps. At the first step, we found which of the drug pairs are associated with selected PTs. At the second step we used LASSO logistical regression [35] to estimate the potential synergistic and additive DDIs that are associated with the drug pairs that were selected in step 1. At this step, noninteracting drug pairs were also determined. At the third step, we integrated the obtained data on different PTs into single ADEs to create datasets with positive and negative examples of DDI-induced ADEs (see Table 1).

#### Step 1. Identification of the association between drug pairs and PTs

A proportional reporting ratio (PRR) was used to determine the drug pairs that are associated with each PT. PRR is calculated as follows:

$$PRR=\frac{A(B+D)}{B(A+C)}$$ (1)

The value A is a number of the SRs where both the drug pair and PT are mentioned; B is a number of SRs where PT is mentioned, but the drug pair is not mentioned; C is a number of SRs where the drug pair and other PTs are mentioned; and D is a number of SRs where the PT and drug pair are not mentioned.

According to previously published criteria [26, 28], we considered a relationship between the drug pair and PT if PRR ≥ 2, A ≥ 3 and chi-square ≥ 4. The selected associations were used at the next step of analysis.

#### Step 2. Identification of synergistic and additive DDIs

We identified synergistic and additive pairwise DDIs that are associated with each PT by using LASSO logistic regression with propensity scores (PSs). The method is described in detail in the original publication [35].

Briefly, PS is a conditional probability of being exposed to a drug that is calculated for each SR. This probability depends on the patient’s diseases and, indirectly, on co-administered drugs. The PS indirectly reflects the influence of human diseases and co-administered drugs on the development of ADE, and, thus, allows for the filtering of many false positive drug-ADE associations. We calculated the PSs for each drug-SR pair based on the top 100 co-administered drugs and the top 100 most relevant drug indications. The relevance of co-administered drugs and indications of a drug were measured by a phi correlation coefficient, which is a square root of ratio of the corresponding chi-squared statistic to the total number of SRs [42].

The final values of the PSs were calculated by using the following logistic regression:

$$PS=logit(P(drug=1))=\alpha +\sum_{i=1}^{100}\beta_{i}{In}_{i}+\sum_{j=1}^{100}\gamma_{j}{Dr}_{j}$$ (2)

In formula (2), the values In_i_ and Dr_j_ are the indication and co-administered drug with relevance ranks i and j.

Next, we used LASSO logistic regression to estimate the probability of PT for each SR that depends on the presence of two drugs in SR, their possible interaction, and the corresponding PSs as follows:

$$logit(P(PT=1))=\beta_{0}+\beta_{1}{PS}_{1}+\beta_{2}{PS}_{2}+\beta_{3}{Drug}_{1}+\beta_{4}{Drug}_{2}+\beta_{5}{Drug}_{1}*{Drug}_{2}+\lambda {\left|\beta \right|}_{1}$$ (3)

In formula (3), PS_1_ and PS_2_ are PSs for drug_1_ and drug_2_, |β|_1_ is l_1_ norm of coefficients, and λ is a tuning parameter of regularization. Parameter λ was determined through a 3-fold cross-validation procedure using all SRs. The potential synergistic and additive DDIs that are associated with PTs were determined based on β_3_, β_4_ and β_5_ coefficients:

- *synergistic DDI* for drug pair-PT association was considered if β_5_ was more than 0;
- *additive DDI* for drug pair-PT association was considered if β_5_ equals 0, β_3_ and β_4_ were more than 0, and drug_1_, drug_2_ have known links to the corresponding ADE in datasets from our previous study [37].
- *absence of DDI* for the drug pair-PT association was considered if either β_3_ or β_4_ were less or equal to 0, and β_5_ was less or equal to 0. Additionally, we considered the absence of DDIs if the corresponding drug pair-PT association was not determined at step 1 (the condition PRR ≥ 2, A ≥ 3 and chi-square ≥ 4 was not true), but the drug pair itself was mentioned in at least 500 SRs with other PTs. This threshold was chosen because it allows achieving the highest accuracy of classification using predicted drug-target interactions as descriptors and Random Forest.

#### Step 3. Integration of data on different PTs

To create final datasets with the information on DDI-induced ADEs, we integrated data on the PTs as follows:

- The drug pair was considered to be potentially “positive” according to the corresponding ADE if it was linked to at least two main PTs, or at least to one main and one supporting PT at step 2 of the analysis.
- The drug pair was considered to be potentially “negative” according to the corresponding ADE if it was linked to neither of the PTs that are associated with this ADE. Additionally, we removed from this category those drug pairs in which both drugs are ADE-causing, according to data from our previous study [37], as potentially false negatives.

As a result, datasets for the five cardiovascular ADEs were created (see Results and Discussion, Table 1).

### Validation of datasets of drug pairs with information on five ADEs

Since the datasets on five cardiovascular ADEs were created using analysis of SRs, they may still contain false positive and false negative associations between drug pairs and corresponding effects, thus, datasets have to be validated before performing further analysis. For this purpose we used inference scores (ISs) from Comparative Toxicogenomics Database [38]. ISs were calculated based on known interactions of drugs with human genes which have links to corresponding diseases in literature. ISs reflect the degree of similarity between drug–gene–disease networks and a similar scale-free random network. The higher the score, the more likely the inference network has atypical connectivity (see original publication [38] for details) and the higher the probability of possible relationship between drug and disease. If drug is not known to cause ADE according to data from our previous study (see above) [37] we took IS from Comparative Toxicogenomics Database for corresponding disease; however if the drug is known to cause ADE we took the maximal value of ISs among all drugs. It was done because many drugs, which have description of cardiovascular ADEs in “Boxed Warning” and “Warnings and Precautions” sections of drug labels, demonstrate low ISs due to insufficient information on target genes in literature. To describe pairs of drugs with corresponding ISs we used sums of the scores of individual drugs. We calculated AUC values for each of the five datasets based on ISs for corresponding diseases (ventricular tachycardia, myocardial infarction, ischemic stroke, arterial hypertension and cardiac failure). We proposed that the values of AUC reflect the quality of datasets.

### Prediction of drug-target interactions

Interactions of individual drugs with human proteins were predicted by the PASS Targets software [39]. PASS (Prediction of Activity Spectra for Substances) [43–45] can be used for the prediction of various types of biological activities and is associated with several hundred success stories of its practical application, with experimental confirmation of the prediction results [45, 46]. It uses Multilevel Neighborhoods of Atoms (MNA) descriptors and the Bayesian approach and is available as a desktop program as well as a freely available web service on the Way2Drug platform (http://www.way2drug.com/PASSOnline/) [47]. PASS Targets is a special version of PASS that is based on training data from the ChEMBL database (https://www.ebi.ac.uk/chembl/) and allows for predicting interactions with 1553 human protein targets with an average AUC 0.97 and a minimal AUC 0.85 [39]. The full list of human targets is presented in S3 Table.

PASS provides two estimates of probabilities for each target of a chemical compound: The Pa probability to interact with a target, and the Pi probability to not interact with a target. If a compound has Pa > Pi, it can be considered as interacting with the target. The larger the Pa and Pa−Pi values, the greater the probability of obtaining an activity against a target in the experiment. In this study, we used a threshold Pa>0.3 for the estimation of protein targets of drugs from the top 10 scored drug pairs potentially causing ventricular tachycardia (see the last section of the Results and Discussion).

We used sums and absolute values of differences of Pa/(Pa+Pi) values, calculated by PASS for individual drugs, to obtain corresponding values for pairs of drugs. Thus, each drug pair was described by a vector of 3106 values, which were further used as descriptors for the creation of classification models (see below).

### Creation of classification models for DDI-induced cardiovascular ADEs

Classification models for the prediction of five DDI-induced cardiovascular ADEs were created by the Random Forest method. We used the RandomForest function from “RandomForest” R package (https://cran.r-project.org/web/packages/randomForest/) for this purpose. All arguments of this function were set to default. Since the training sets were imbalanced (see Table 1) which is a problem for the creation of accurate classification models we used multiple under-sampling procedure when majority class of the training set was randomly sampled up to the size of the minority class. This process was repeated multiple times, and prediction probabilities from multiple models were averaged.

The applicability domain of the obtained models was determined by the local (Tree) approach, which was described earlier [40].

The accuracy of created models was determined by a 5-fold cross validation procedure according to the “compound out” approach, wherein each drug pair in the test set must contain at least one drug that is absent in all drug pairs of the training set [41].

The accuracies of the models for ventricular tachycardia and arterial hypertension were also estimated on two external test sets generated based on information from DrugBank (https://www.drugbank.ca/) database. The database contains some data on known DDIs that lead to ventricular tachycardia (or prolongation of the QT interval on an electrocardiogram) and arterial hypertension. These DDIs were extracted from drug labels and scientific publications by DrugBank team. We used this data as positive examples to create external tests sets. To create negative examples, we randomly generated drug pairs in the amounts equal to positive examples. We did not include as negative examples those pairs, where both individual drugs cause corresponding ADE according to data from our previous study [37] (see above), as potentially false negatives.

## Supporting information

S1 Table Datasets with information of DDI-induced cardiovascular ADEs. (XLSX)

S2 Table Information about cardiovascular ADEs of individual drugs used in the study. (XLSX)

S3 Table The list of human protein targets predicted by PASS Targets software. Numbers of active compounds in the training set as well as the AUC values that were obtained by leave-one-out cross-validation are given.
(XLSX)
